# Fructose Induces Insulin Resistance of Gestational Diabetes Mellitus in Mice via the NLRP3 Inflammasome Pathway

**DOI:** 10.3389/fnut.2022.839174

**Published:** 2022-04-12

**Authors:** Yao Liu, Yuanhuan Wei, Lanlan Wu, Xiaoping Lin, Ruifang Sun, Hengying Chen, Siwen Shen, Guifang Deng

**Affiliations:** ^1^Department of Clinical Nutrition, Union Shenzhen Hospital of Huazhong University of Science and Technology, Shenzhen, China; ^2^Guangdong Provincial Key Laboratory of Tropical Disease Research, Department of Nutrition and Food Hygiene, School of Public Health, Southern Medical University, Guangzhou, China; ^3^Injury Prevention Research Center, Shantou University Medical College, Shantou, China

**Keywords:** gestational diabetes mellitus, insulin resistance, fructose, nucleotide-binding domain-like receptor protein 3 inflammasome, nuclear factor-κappa B

## Abstract

**Background:**

Insulin resistance (IR), which is affected by dietary factors, is the main pathology underlying of gestational diabetes mellitus (GDM). Fructose (Fru), a sugar found in fruits, honey, and food sweeteners, has been reported to induce IR and inflammation. This study explored the effects and mechanisms of Fru on IR of GDM in pregnant and postpartum mice and their offspring.

**Methods:**

The 6-week-old female C57BL/6J mice were randomly divided into control (Chow) and fructose (Fru) groups, with the latter receiving 20% (w/v) Fru in drinking water from 2 weeks before pregnancy to the end of pregnancy. The effects of Fru on IR and inflammation were determined using serum parameters, glucose metabolism tests, immunohistochemistry, and western blotting.

**Results:**

Compared with the Chow group mice, pregnant mice treated with Fru exhibited greater gestational weight gain, higher fasting blood glucose and insulin concentrations, and a higher homeostasis model of assessment (HOMA) for IR index, but a lower HOMA for insulin sensitivity index. Treatment with Fru also increased the concentrations of interleukin-6 (IL-6), tumor necrosis factor-α (TNF-α), IL-17, and C-reactive protein in sera and the expression of IL-6, TNF-α, IL-17, and IL-1β mRNA in liver tissues of pregnant mice. Both CD68 and IL-1β positive cell were increased in Fru-treated mice compared with in Chow mice. Fru treatment also promoted IR and inflammation in mice at 4 weeks after delivery and in offspring mice. Mechanistically, Fru promoted the nuclear translocation of nuclear factor-kappa B (NF-κB) p65 to activate the nucleotide-binding domain-like receptor protein 3 (NLRP3) inflammasome.

**Conclusions:**

Exposure to Fru before and during pregnancy induced IR in pregnant mice, which continued at 4 weeks postpartum and affected the offspring. The effects of Fru may be associated with activation of the NF-κB–NLRP3 pathway.

## Introduction

Gestational diabetes mellitus (GDM), a type of diabetes that occurs in association with pregnancy, is a common maternal medical complication (1). Between 1991 and 2000, the incidence of GDM among women of different races increased by 16–127% (2), and a recent meta-analysis found that the prevalence of GDM among women in mainland China was 14.8% (95% confidence interval [*CI*]: 12.8–16.7%) (3). GDM endangers maternal health and fetal development. Specifically, in pregnant women, GDM is associated with a higher incidence of hypertensive disorders, pregnancy complications, premature delivery, lipid metabolic abnormalities, postpartum hyperglycemia, and puerperal infection (4). In addition, GDM can significantly increase the risks of obesity and impaired glucose tolerance in offspring (5). Although insulin resistance (IR) is the main cause of GDM, the underlying pathogenic mechanism remains unclear. Therefore, the study of IR during pregnancy is expected to reveal why some pregnant women develop GDM and to improve the maternal and neonatal outcomes associated with this disorder.

Recent in-depth studies of the insulin signaling pathway have revealed the importance of inflammation in abnormal glucose metabolism. For example, inflammatory factors such as tumor necrosis factor α (TNF-α), interleukin-6 (IL-6), and C-reactive protein (CRP) have been reported to contribute to IR in GDM (6, 7). The nucleotide-binding oligomerization domain-like receptor (NLRP3) inflammasome is a macromolecular polyprotein complex composed of NLRP3, the apoptosis-associated speck-like protein containing caspase recruitment domain (ASC) and cysteinyl aspartate specific proteinase-1 (caspase-1) (8). The interaction of pathogen-related molecular patterns (PAMPs) or damage-related molecular patterns (DAMPs) with caspase-1 activates the NLRP3 inflammasome, causing immune cell to secrete mature IL-1β and IL-18 to mediate an inflammatory response (8). Studies have revealed a close relationship between the NLRP3 inflammasome and the development of inflammatory diseases such as type 2 diabetes mellitus (T2DM), Alzheimer's disease, atherosclerosis, and gout (9–11). The NLRP3 inflammasome also plays an important regulatory role in IR, as demonstrated by a study in which NLRP3 knockout significantly improved high-fat diet-induced IR of mice (12). Increasingly, evidence suggests that activation of the NLRP3 inflammasome contributes to the development of T2DM (9, 13). However, no studies have been conducted to investigate whether the NLRP3 inflammasome plays a role in the development of GDM.

Fructose (Fru) is a type of sugar found in fruits and honey. It is mainly available as a component of the disaccharide sucrose, which is degraded by intestinal enzymes to release free Fru and glucose (14). High-fructose corn syrup (HFCS), which is used as a sweetener in soft drinks, pastries, desserts, and various processed foods, is another major source of Fru (14). Large increases in the HFCS consumption worldwide have led to sharp spikes in the global prevalence of both diabetes mellitus and obesity (15). However, a potential causal relationship between Fru and diabetes mellitus remains highly controversial. Fru has been shown to potentially reduce postprandial fluctuations in the blood glucose concentration when compared with isocaloric starch; consequently, Fru has been used as an alternative sweetener by people with diabetes mellitus (16). However, several recent epidemiological studies have found that high Fru intake is closely related to the incidence of obesity, diabetes mellitus, fatty liver, and other metabolic disorders (17, 18). Choo et al. showed that high Fru intake significantly increased the fasting blood glucose (FBG) and fasting insulin (FINS) concentrations (19). Most recently, a study of middle-aged and older Chinese adults revealed an independent association between elevated fasting serum Fru concentrations and an increased incidence of T2DM (20), suggesting that earlier studies showing the beneficial effects of Fru in people with diabetes mellitus might have neglected the long-term effects of Fru on IR. Moreover, few studies have explored whether Fru intake is associated with the increasing incidence of GDM. Accordingly, the potential effects of a high Fru intake on IR and consequent GDM during and after pregnancy need to be explored further.

In this study, we explored the effects of Fru on IR in pregnant, postpartum, and offspring mice. We further investigated whether the effects of Fru on IR associated with GDM are mediated through the NLRP3 signaling pathway.

## Materials and Methods

### Animal Model

The Guangdong Medical Laboratory Animal Center (Guangzhou, China) provided 6-week-old C57BL/6 mice (40 females, 20 males). All animal experiments were performed in compliance with the Guide of the Medical Ethics Committee of Shenzhen Nanshan District People's Hospital (permit number: [2019]072644). The female mice were randomly divided into two groups (*n* = 20/group): the Chow group (Chow) was fed an AIN-93G diet (LAD3001G, Trophic Animal Feed High-tech Co., Ltd., Haicheng, China) and untreated drinking water, while the Fru group was fed an AIN-93G diet, and water supplemented with 20% (w/v) Fru. The male mice were fed with an AIN-93G diet and untreated drinking water *ad libitum*. After a 2-week intervention, the male and female mice were placed in the same cages at a 1:2 ratio for 24 h. Successful fertilization of the female mice was confirmed by the formation of a vaginal plug. Fru supplementation was continued during pregnancy, and the body weights of the mice were recorded on day 0, 3, 10, and 18 of pregnancy. On day 21 of pregnancy, half of the mice in each group were sacrificed, and blood samples and liver tissues were collected. The other mice were allowed to deliver offspring normally and sacrificed at 4 weeks postpartum after being fed an AIN-93G diet and untreated drinking water. Samples were collected from all mice.

### Measurement of Serum Glucose Parameters

The FINS concentration was measured using an ultrasensitive mouse insulin enzyme-linked immunosorbent assay (ELISA) kit (ALPCO, Salem, NH, USA). The FBG concentration was determined using a blood glucose assay kit (Solarbio, Beijing, China) based on the glucose oxidase method. The homeostasis model of assessment (HOMA) for IR index (HOMA-IR), HOMA for islet β-cell function (HOMA-β%), and HOMA for insulin sensitivity index (HOMA-ISI) were calculated using the following formulas:

HOMA-IR = FBG × (FINS/22.5); HOMA-β% = 20 × FINS/(FBG – 3.5); and HOMA-ISI = ln[1/(FBG × FINS)].

### Measurement of Serum Inflammatory Factor Concentrations

The serum concentrations of IL-6 (#AZ0523), IL-17 (#AZ0549), TNF-α (#AZ0625), CRP (#AZ0635), IL-1β (#AZ0513), and IL-18 (#AZ0545) were measured using respective ELISA kits according to the manufacturer's instructions (Applygen, Beijing, China).

### Immunohistochemistry

The liver tissues were embedded in paraffin, sectioned into 10-μm-thick slices and stored at −20°C. The sections were incubated with 3% H_2_O_2_ at 37°C for 5–10 min to block endogenous peroxidases and then rinsed with distilled water and incubated twice in phosphate-buffered saline (PBS) for 5 min. After blocking with 5% goat serum in PBS for 10 min at 37°C, the slices were incubated with a primary antibody specific for CD68 (Cell Signaling Technology, Danvers, MA, USA) for 12–16 h at 4°C. The sections were washed with PBS and then incubated with a horseradish peroxidase polymer in the dark for 20 min at room temperature. After washing again with PBS, the sections were treated with a streptavidin–biotin complex for 30 min. Next, the sections were treated with a chromogenic agent (3,3'-diaminobenzidine) and observed under a microscope. After washing under flowing water, the sections were counterstained with hematoxylin for 2 min and then washed, dehydrated, transparent, and sealed.

### Real-Time Quantitative Reverse-Transcriptase Polymerase Chain Reaction (qRT-PCR)

Total RNA was isolated from the liver tissues using TRIzol (Invitrogen, Carlsbad, CA, USA). Next, the RNAs were reverse-transcribed to yield cDNA using the PrimeScript RT reagent kit (Takara, Shiga, Japan). The SYBR Premix ExTaqII (Tli RNase H Plus) kit (Takara) was used to perform quantitative real-time polymerase chain reaction (qRT-PCR) on a Vii7 system (Applied Biosystems, Waltham, MA, USA). All primers used (Sangon Biotech, Shanghai, China) are listed in the Supplementary Table 1.

### Western Blotting

Proteins were extracted from liver tissues and prepared for Western blotting, as described in our previous publication (21). The following antibodies were purchased from Cell Signaling Technology: rabbit anti-NF-κB p65 (Cat# 3033S), anti-phosphorylated (phospho)-NF-κB p65 (Cat# 8242S), anti-IκBα (Cat# 4812S), anti-phospho-IκBα (Cat# 2859S), anti-NLRP3 (Cat# 15101S), anti-ASC (Cat# 67824S), and cleaved caspase-1 (Cat# 89332S). The following antibodies were purchased from Proteintech (Rosemont, IL, USA): anti-caspase-8 (Cat# 13423-1-AP), anti-FAS-associated death domain protein (FADD, Cat# 14906-1-AP), anti-caspase-1p20 (Cat# 22915-1-AP), anti-IL-1β (Cat# 16806-1-AP), anti-histone H3 (Cat# 17168-1-AP) and anti-GAPDH (Cat# 10494-1-AP). An anti-procaspase-1 antibody (Cat# ab238972) was purchased from Abcam (Cambridge, MA, USA).

### Glucose Metabolism Tests

Mice underwent oral glucose tolerance tests (OGTT) after fasting for 12 h. Each mouse was injected intraperitoneally with 2 g glucose/kg body weight (bw; 10 μl/g bw). The glucose (Fresenius-kabi SSPC, Beijing, China) solution was prepared in normal saline. Tail vein blood was sampled to measure the glucose concentration at 0, 30, 60, 90, and 120 min postinjection.

Mice were subjected to insulin tolerance tests (ITT) after fasting for 5 h. Each mouse was intraperitoneally injected with 0.50 IU insulin/kg bw (10 μL/g bw) (Tonghua Dongbao Pharmaceutical Co., Ltd., Jilin, China). Tail vein blood was sampled to measure the glucose concentration at 0, 30, 60, 90, and 120 min Post-injection.

### Caspase-1 Activity Assay

Caspase-1 enzymatic activity was measured according to the manufacturer's specification by using caspase assay kit (#BC3810, Solarbio, Beijing, China). The optical density (OD) value was measured at 450 nm.

### Immunofluorescence

The sections of liver tissues were fixed in 4% paraformaldehyde for 30 min and then permeabilized in 0.05% TritonX-100 for 10 min. The sections were stained with primary antibodies against CD68 (CST) and IL-1β (CST) at 4°C overnight, followed by the blocking with 1% BSA for 1 h. After washing with PBS three times, sections was stained with FITC anti-mouse IgG (Proteintech) and Cy3 anti-rabbit IgG (Proteintech) for 1 h. A laser scanning confocal microscope (Leica, Wetzlar, Germany) was used to capture the images.

### Statistical Analysis

The data are shown as means ± standard deviations (*SD*s) and were compared using Student's *t*-test (two-tailed test). SPSS 22.0 software (IBM Inc. Chicago, IL, USA) was used for statistical analysis. A *p-*value ≤ 0.05 was considered to indicate statistical significance.

## Results

### Effects of Fru on the Progression of GDM in Mice

**Figure 1:**
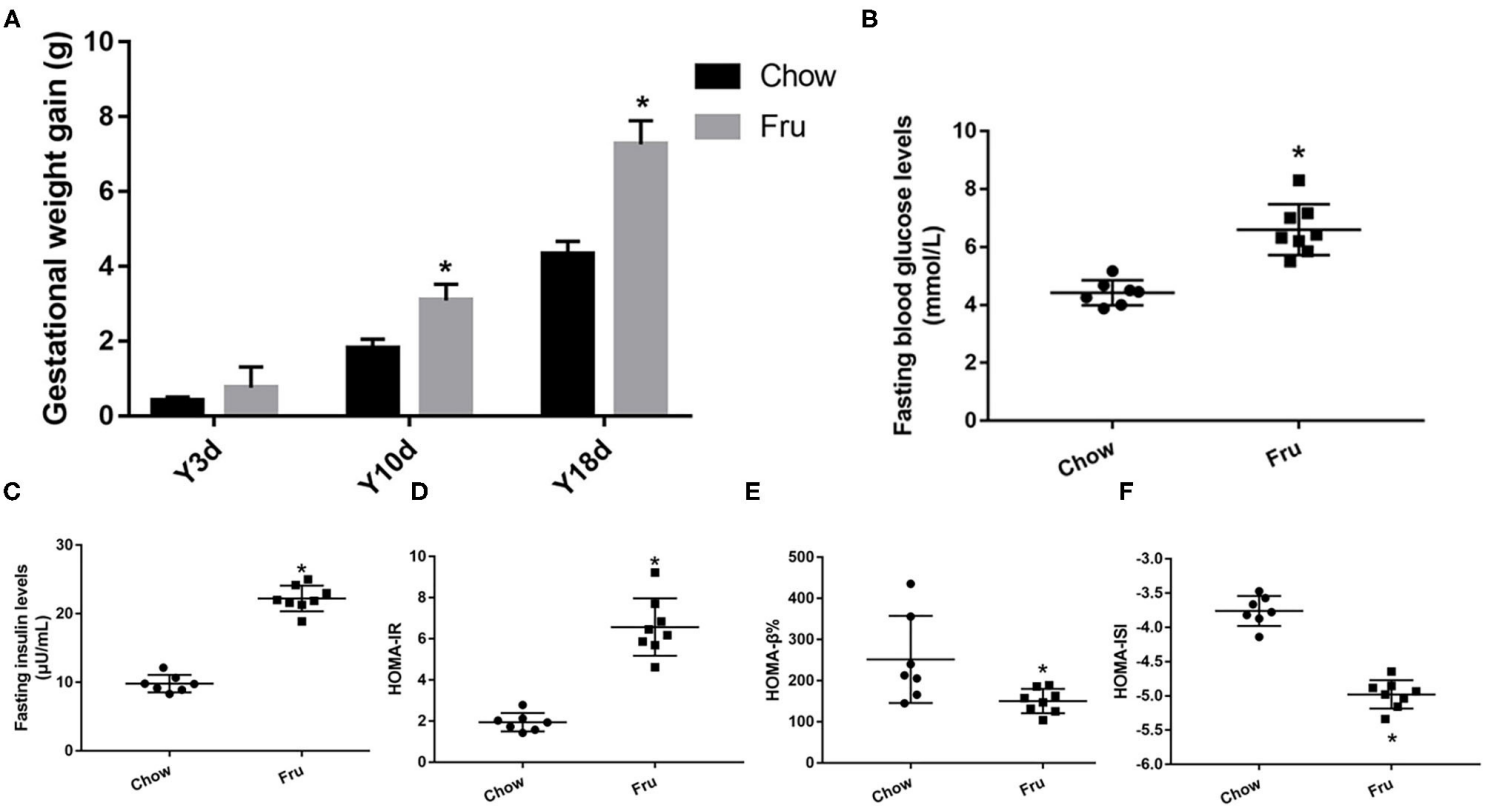
Fru induces IR of GDM in pregnant mice. Mice were drunk with 20% Fru or normal water. On the 21st day of pregnancy, half of the mice in each group were sacrificed (*n* = 9). **(A)** Gestational weight gain. **(B)** Fasting serum glucose levels. **(C)** Fasting serum insulin levels. **(D)** HOMA-IR. **(E)** HOMA-β%. **(F)** HOMA-ISI. Data are shown as the means ± SD. *p < 0.05.

The animal experimental protocol is shown in Supplementary Figure 1. The Fru intervention began 2 weeks before pregnancy and continued throughout pregnancy. Compared with mice in the Chow group, mice in the Fru group exhibited an increase in weight gain during pregnancy (Figure 1A). To assess the effects of Fru on glucose metabolism, we measured the FBG and FINS concentrations and used these values to calculate HOMA-IR, HOMA-ISI, and HOMA-β%. As expected, mice in the Fru group had significantly higher FBG (4.42 ± 0.164 vs. 6.413 ± 0.278, *p* < 0.01) and FINS (9.81 ± 0.48 vs. 22.25 ± 0.66, *p* < 0.01) concentrations and a significantly higher HOMA-IR (1.95 ± 0.17 vs. 6.36 ± 0.46, *p* < 0.01; Figures 1B–D). However, the Fru group had significantly lower HOMA-β% and HOMA-ISI values than the Chow group (Figures 1E,F all *p-*values < 0.05). These results indicated that mice subjected to the Fru intervention were more likely to develop IR during pregnancy.

### The Effects of Fru on Inflammation in Mice Were Mediated by the Activation of NF-κB and the NLRP3 Inflammasome

**Figure 2:**
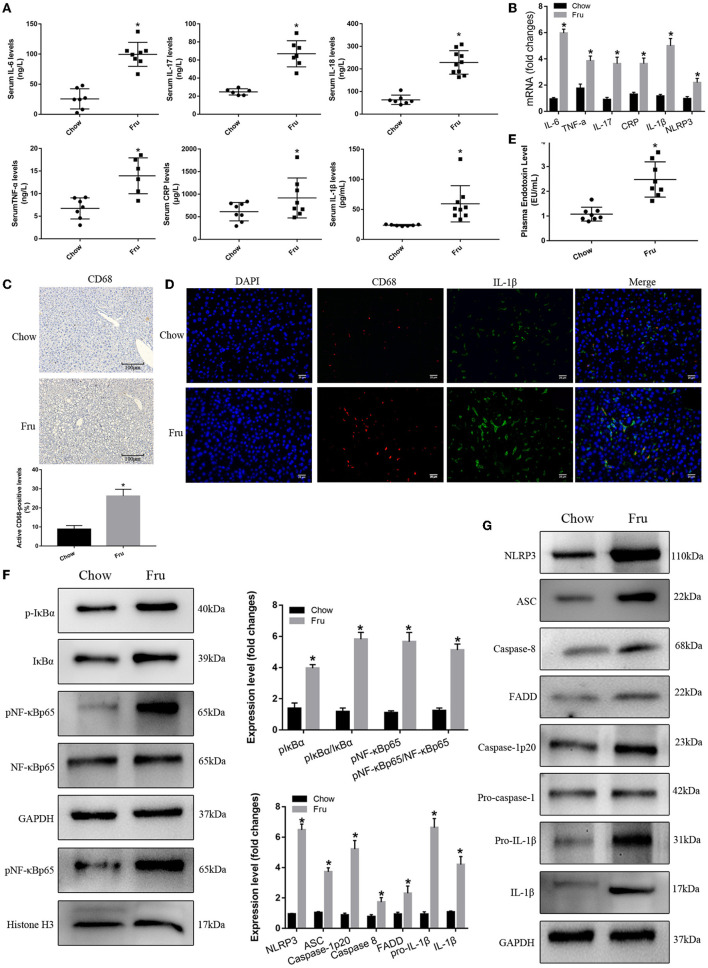
Fru promotes inflammation by inducing NF-κB and NLRP3 inflammasome activation in pregnant mice. Mice were drunk with 20% Fru or normal water. On the 21st day of pregnancy, half of the mice in each group were sacrificed. **(A)** Serum IL-6, IL-17, TNF-α, CRP, IL-1β, and IL-18 concentrations (*n* = 7). **(B)** The mRNA levels of IL-6, IL-17, TNF-α, CRP, IL-1β, and NLRP3 levels in liver of mice (*n* = 6). **(C)** CD68 positive macrophages determined by immunohistochemistry staining in liver of mice (*n* = 5). **(D)** Representative images of co-expression of CD68 (red) and IL-1β (green) in liver tissue of mice. **(E)** Plasma endotoxin level (*n* = 7). **(F)** Respective Western blots showing pIκBα, IκBα, pNF-κBp65, and NF-κB p65, and nuclear pNF-κBp65 in liver of mice (*n* ≥ 3). **(G)** Respective Western blots showing NLRP3, ASC, caspase-8, FADD, caspase-1p20, pro-IL-1β, and IL-1β in liver of mice (*n* ≥ 3). Data are shown as the means ± SD. *p < 0.05.

We next examined the effect of Fru on inflammation, a key factor in IR associated with GDM. As shown in Figure 2A, the serum concentrations of the inflammatory cytokines IL-6, TNF-α, IL-17, CRP, IL-1β, and IL-18 were significantly elevated in the Fru group compared to the Chow group. Analysis of the corresponding mRNA levels revealed the same trends (Figure 2B). Immunohistochemical analysis revealed an obvious increase in the infiltration of CD68-positive cells after the Fru intervention (Figure 2C). Both CD68 and IL-1β levels were elevated in Fru-treated mice compared with in Chow mice (Figure 2D).

Next, we determined whether Fru enhances inflammation by regulating the activation of NF-κB and the NLRP3 inflammasome. First, we confirmed that Fru was associated with an increase in the plasma endotoxin concentration (Figure 2E). Next, we performed Western blotting to detect the levels and activation statuses of proteins upstream and downstream of the NF-κB–NLRP3 signaling pathway. We observed a dramatic increase in the level of phosphorylated IκBα in the Fru group, which was followed by the activation of NF-κB as indicated by increases in the levels of phosphorylation and nuclear translocation of NF-κB. Increased levels of caspase-8 and FADD were also observed in mice in the Fru group. Furthermore, treatment with Fru was associated with remarkable increases in the levels of the active isoform of caspase-1p20 and the mature form of IL-1β, which were accompanied by increases in the levels of NLRP3, ASC, and caspase-1 (Figures 2F,G). Taken together, these results suggest that Fru induces inflammation associated with IR in mice by activating the NF-κB–NLRP3 signaling pathway.

### Effects of Fru on Glycolipid Metabolism and Inflammation in Mice at 4 Weeks Postpartum

**Figure 3:**
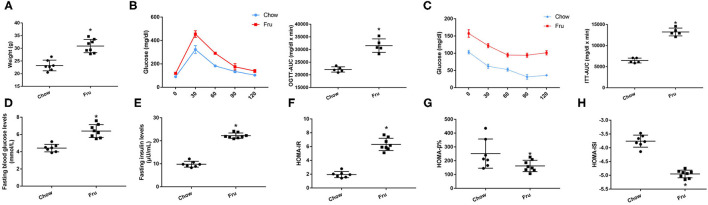
Fru induces IR of mice at 4 weeks postpartum. Mice were drunk with 20% Fru or normal water from 2 weeks before pregnancy to whole pregnancy. After delivery, mice were fed with normal chow diet and drinking water for 4 weeks (*n* = 7). **(A)** Weight of mice. **(B)** Oral glucose tolerance tests and the AUC of OGTT. **(C)** Insulin tolerance tests and the AUC of ITT. **(D)** Fasting serum glucose levels. **(E)** Fasting serum insulin levels. **(F)** HOMA-IR. **(G)** HOMA-β%. **(H)** HOMA-ISI. Data are shown as the means ± SD. *p < 0.05.

**Figure 4:**
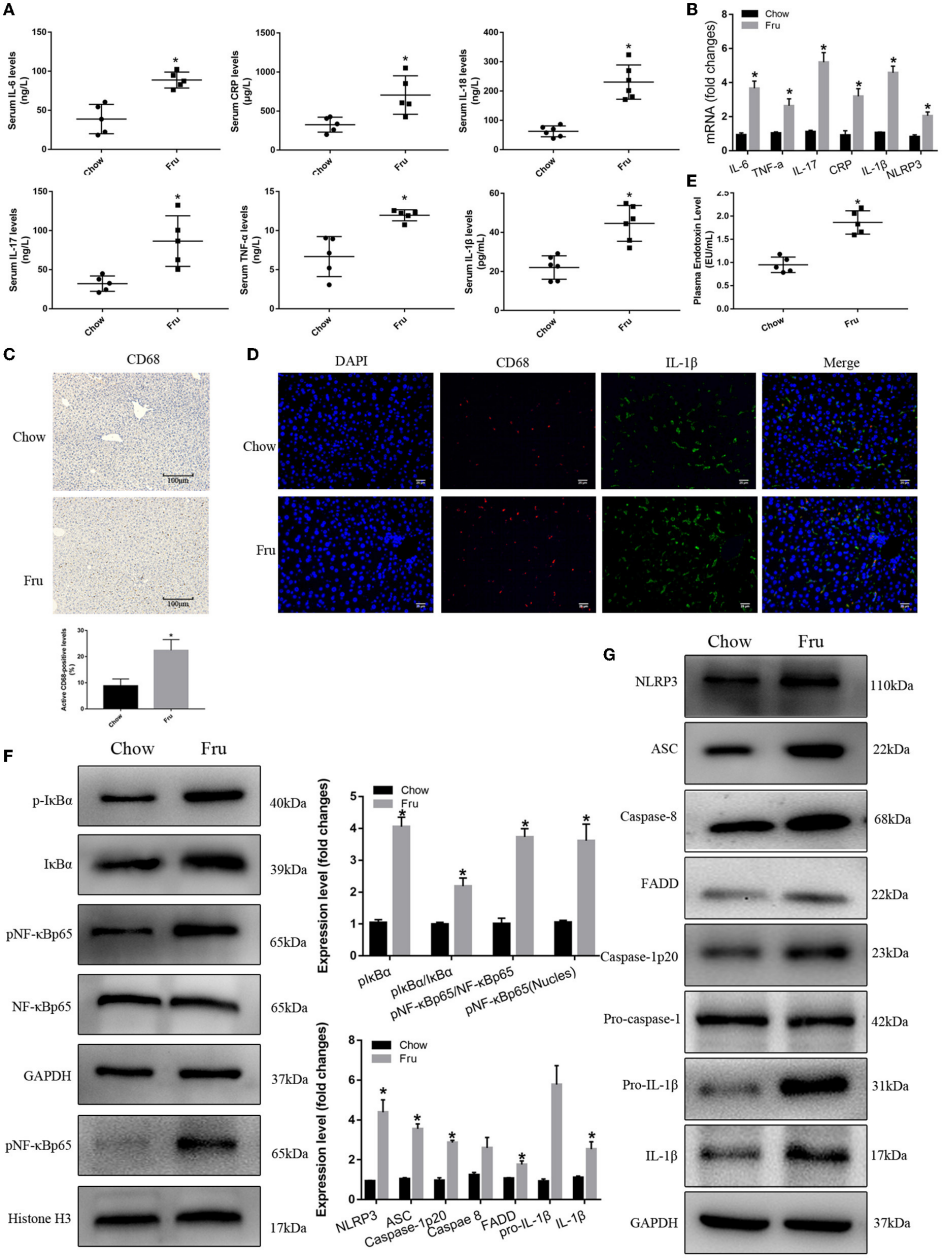
Fru promotes inflammation of mice at 4 weeks postpartum. Mice were drunk with 20% Fru or normal water from 2 weeks before pregnancy to whole pregnancy. After delivery, mice were fed with normal chow diet and drinking water for 4 weeks. **(A)** Serum IL-6, IL-17, TNF-α, CRP, IL-1β, and IL-18 concentrations (*n* = 6). **(B)** The mRNA levels of IL-6, IL-17, TNF-α, CRP IL-1β, and NLRP3 levels in liver of mice. **(C)** CD68 positive macrophages determined by immunohistochemistry staining in liver of mice (*n* = 5). **(D)** Representative images of co-expression of CD68 (red) and IL-1β (green) in liver tissue of mice. **(E)** Plasma endotoxin level (*n* = 6). **(F)** Respective Western blots showing pIκBα, IκBα, pNF-κBp65, and NF-κB p65, and nuclear pNF-κBp65 in liver of mice (*n* ≥ 3). **(G)** Respective Western blots showing NLRP3, ASC, caspase-8, FADD, caspase-1p20, pro-IL-1β, and IL-1β in liver of mice (*n* ≥ 3). Data are shown as the means ± SD. *p < 0.05.

We also explored the effects of Fru on glycolipid metabolism and inflammation in mice after gestation and delivery, and the results are presented in (Figures 3, 4). At 4 weeks postpartum, mice in the Fru group had a higher body weight than those in the Chow group (Figure 3A). Additionally, the increases in OGTT-AUC and ITT-AUC were 1.35 and 2.22 times greater, respectively, in the Fru group than in the Chow group (Figures 3B,C). We also observed higher FBG and FINS concentrations and a higher HOMA-IR value but lower HOMA-β% and HOMA-ISI values in the Fru group than in the Chow group ((Figures 3D–H).

Fructose also promoted inflammation in mice at 4 weeks postpartum. Specifically, mice in the Fru group exhibited 128, 169, 79, 117, 102, and 268% increases in the serum concentrations of IL-6, IL-17, TNF-α, CRP, IL-1β, and IL-18, respectively, and these increases were significant (Figure 4A). The results of qRTPCR showed that the corresponding mRNA levels revealed the same trends (Figure 4B). Mice in the Fru group also exhibited enhanced hepatic CD68 and IL-1β protein expression (Figures 4C,D). When compared to the Chow group, mice in the Fru group had a 96% increase in the plasma endotoxin concentration (Figure 4E). We observed significantly enhanced NF-κB and NLRP3 inflammasome activity in mice at 4 weeks postpartum (Figure 4F). Compared with mice in the Chow group, mice in the Fru group exhibited increased expression of NLRP3, ASC, and caspase-1, and subsequently, increased levels of caspase-1p20 and mature IL-1β (Figure 4G).

### Effects of Fru on Glycolipid Metabolism and Inflammation in Offspring Mice

**Figure 5:**
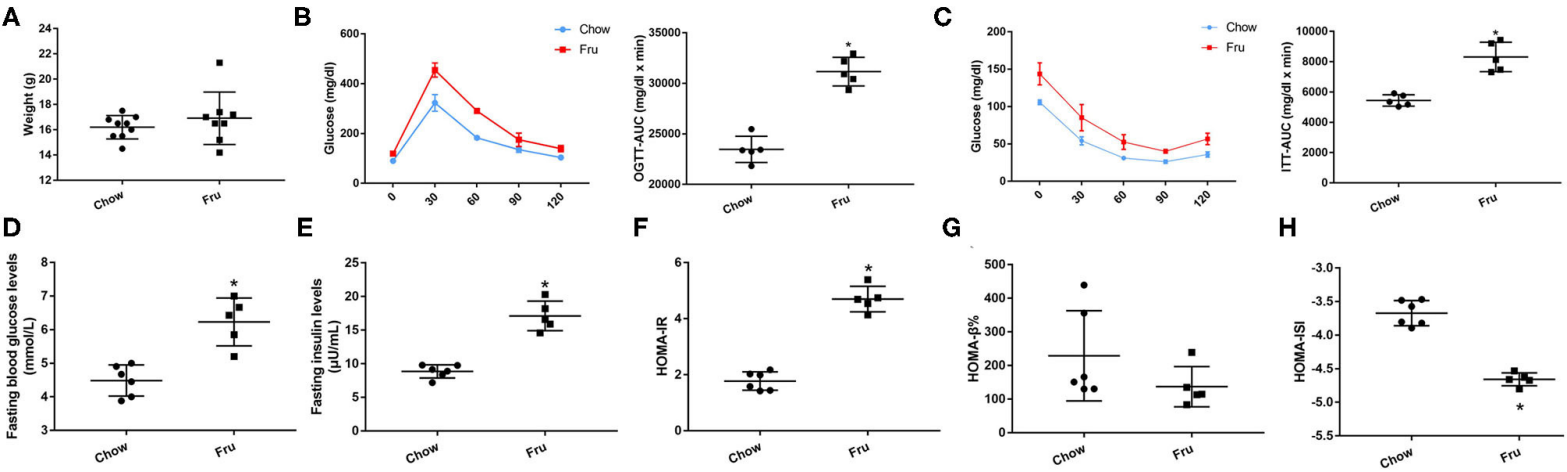
Fru induces IR of 4 weeks old offspring mice. Mice were drunk with 20% Fru or normal water from 2 weeks before pregnancy to whole pregnancy. After delivery, offspring mice were fed with normal chow diet and drinking water for 4 weeks. **(A)** Weight of mice (*n* = 9). **(B)** Oral glucose tolerance tests and the AUC of OGTT (*n* = 6). **(C)** Insulin tolerance tests and the AUC of ITT (*n* = 6). **(D)** Fasting serum glucose levels (*n* = 6). **(E)** Fasting serum insulin levels (*n* = 6). **(F)** HOMA-IR (*n* = 6). **(G)** HOMA-β% (*n* = 6). **(H)** HOMA-ISI (*n* = 6). Data are shown as the means ± SD. *p < 0.05.

Finally, we investigated the effects of Fru on glycolipid metabolism and inflammation in 4-week-old offspring mice. As shown in Figure 5, there was no significant difference in body weight between the two groups. Compared with Chow offspring mice, Fru offspring mice exhibited increases in the OGTT-AUC and ITT-AUC, elevated FBG and FINS concentrations and an increased HOMA-IR value.

**Figure 6:**
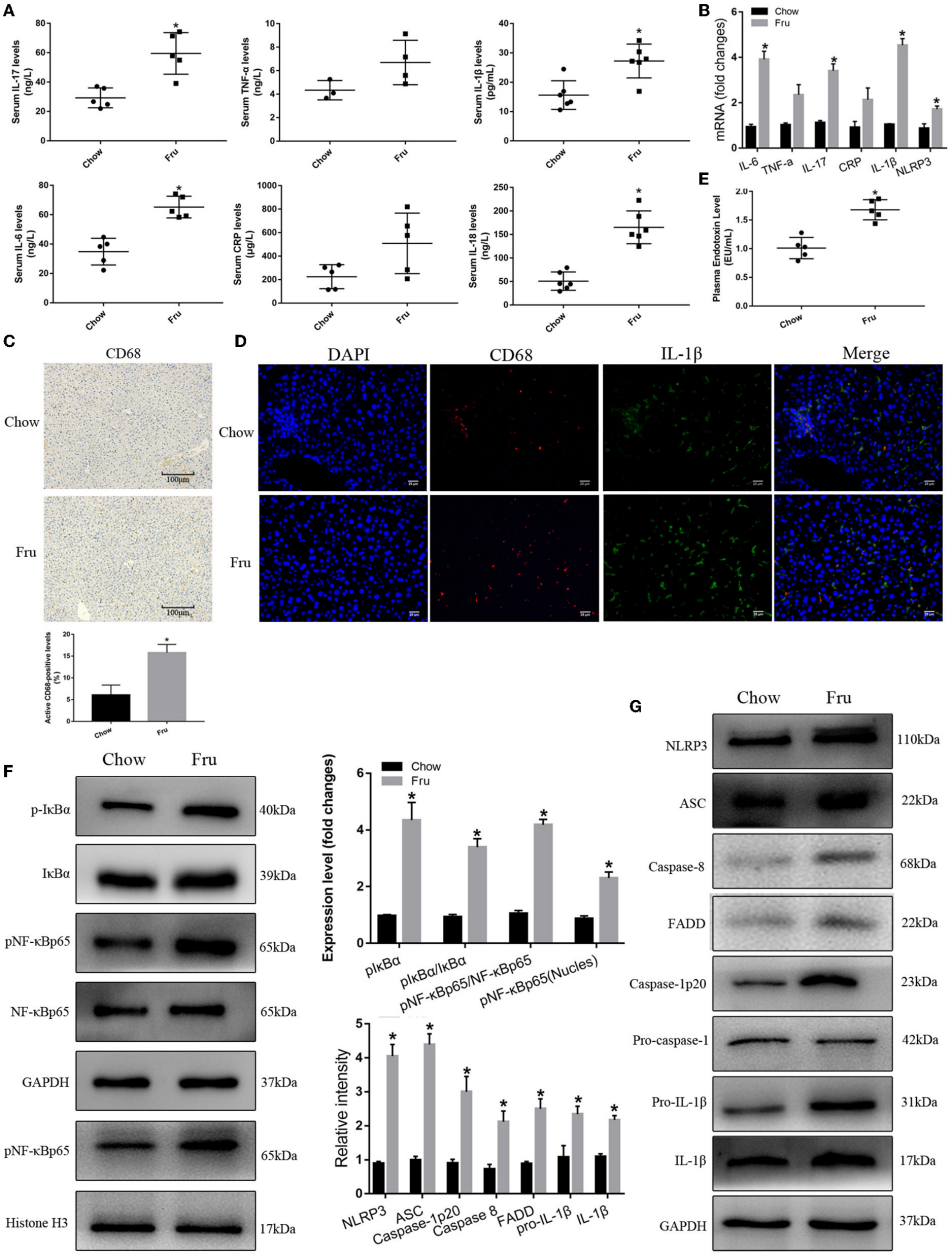
Fru promotes inflammation of 4-weeks-old offspring mice. Mice were drunk with 20% Fru or normal water from 2 weeks before pregnancy to whole pregnancy. After delivery, offspring mice were fed with normal chow diet and drinking water for 4 weeks. **(A)** Serum IL-6, IL-17, TNF-α, CRP, IL-1β, and IL-18 concentrations (*n* = 6). **(B)** The mRNA levels of IL-6, IL-17, TNF-α, CRP IL-1β, and NLRP3 levels in liver of mice. **(C)** CD68 positive macrophages determined by immunohistochemistry staining in liver of mice (*n* = 5). **(D)** Representative images of co-expression of CD68 (red) and IL-1β (green) in liver tissue of mice. **(E)** Plasma endotoxin level (*n* = 6). **(F)** Respective Western blots showing pIκBα, IκBα, pNF-κBp65, and NF-κB p65, and nuclear pNF-κBp65 in liver of mice (*n* ≥ 3). **(G)** Respective Western blots showing NLRP3, ASC, caspase-8, FADD, caspase-1p20, pro-IL-1β, and IL-1β in liver of mice (*n* ≥ 3). Data are shown as the means ± SD. *p < 0.05.

As shown in Figures 6A,B the mRNA levels and serum concentrations of IL-6, IL-17, IL-1β, and IL-18 were significantly higher (*p* < 0.05) in the Fru group than in the Chow group, and offspring in the Fru group exhibited more CD68-positive cell infiltration in the liver than offspring in the Chow group (Figure 6C). Results from immunofluorescence showed that both CD68 and IL-1β expressions were increased in mice of Fru group compared with Chow group (Figure 6D). Offspring in the Fru group also exhibited increases in the plasma concentration of endotoxin (Figure 6E) and activation of the NF-κB–NLRP3 signaling pathway (Figures 6F,G).

## Discussion

This is the first study to demonstrate that high dietary Fru intake can promote the development of IR in pregnant and postpartum mice and their offspring by activating the NF-κB–NLRP3 inflammasome pathway. We found that the treatment with Fru increased the FBG and FINS concentrations and the HOMA-IR and promoted the secretion of proinflammatory cytokines and chemokines and hepatic infiltration by macrophages in pregnant mice. Further findings suggested that the NF-κB–NLRP3 pathway is a key driver of Fru-induced IR in maternal and neonatal mice.

In humans, glucose transporter 5 on the intestinal mucosa transports dietary Fru to the intestinal epithelial cells, where it enters the liver through the portal vein (22). The enzymes involved in Fru metabolism are concentrated in the liver, which is generally considered to be the main site of Fru metabolism (23). However, Soty et al. found that 25% of gluconeogenesis occurs in the small intestine (24). In their study, Rabinowitz et al. fed mice with isotope-labeled Fru and glucose; upon tracking the metabolism of these labeled sugars, the authors demonstrated little fluctuation of the concentration of Fru in the blood (25). Through further metabolomic analysis, Rabinowitz et al. found that about 42% of Fru consumed via the diet was transformed into glucose, 30% was transformed into organic acids, and only 14% entered the liver, indicating that the small intestine was the main site of Fru metabolism (25). At low doses (≤0.5 g/kg), Fru is almost completely metabolized in the small intestine. However, at excess levels that exceed the intestinal absorption and clearance threshold (>1 g/kg), Fru that cannot be cleared in the small intestine is further metabolized by the liver and intestinal flora to produce tricarboxylic acid cycle intermediates, essential amino acids, and short-chain fatty acids (25).

In the long term, excessive Fru intake induces abnormal glucose metabolism and IR. A cohort study revealed that the highest level of exposure to sugar-sweetened beverages, a source of free Fru, was associated with an increased risk of diabetes mellitus relative to the lowest level of exposure (26). A recent prospective Chinese cohort study revealed that a 1-SD increase in the fasting serum Fru concentration was associated with a 35% (95% *CI*: 1.08–1.67) increase in the risk of developing diabetes mellitus (20). In an animal study, the FBG and FINS concentrations increased and the HOMA-ISI decreased after long-term exposure to a high-Fru diet (27). In another study, rats fed a high-Fru diet (66% energy from Fru) for 7 days exhibited an increase in the insulin level and a significant decrease in insulin sensitivity (28). In another rat study, Bezerra et al. found no changes in the expression of the insulin receptor and insulin receptor substrate-1 after exposure to a high-Fru diet for 28 days (29). In that study, however, the hepatic level of tyrosine-phosphorylated insulin receptor decreased significantly to 71% of the control level, the expression of phosphatidylinositol 3-kinase (PI3K) decreased to 84% and the level of phosphorylated tyrosine phosphatase decreased to 79% after insulin stimulation, suggesting that a high-Fru diet can interfere with phosphorylation of the insulin receptor substrate, resulting in IR (29). Recent studies have examined the association between high Fru intake and metabolic diseases such as nonalcoholic fatty liver disease and T2DM. However, few studies have explored the potential association between the intake of Fru and the increasing incidence of GDM.

The association between excessive Fru intake and GDM has been poorly explored in population studies. The Nurses' Health Study II, which reviewed data from 1991 to 2001, found no association between high consumption of whole fruits before pregnancy and an increased risk of GDM (30). A few animal studies have explored the relationship between high Fru intake and GDM development. In pregnant Sprague–Dawley rats, a high-Fru diet induced elevated FBG concentrations during the second trimester of pregnancy (31). Furthermore, Alzamendi et al. highlighted that Fru intake (10% w/v) during pregnancy increased the risk of developing GDM and Pre-eclampsia (32). In another rat study, the administration of Fru in the drinking water throughout gestation resulted in maternal and fetal hypertriglyceridemia and hyperinsulinemia, suggesting the development of IR (33). We similarly found that high Fru intake before and during pregnancy induced IR associated with GDM. In an earlier study, when pregnant rats were exposed to a high-Fru diet, their offspring showed a 140% increase in the plasma insulin concentration relative to that in control offspring at 12 weeks of age (34). A rat study demonstrated that after treatment with 10% w/v Fru in drinking water throughout pregnancy and lactation, the adult offspring (17 weeks old) had an elevated body weight and blood glucose concentration and exhibited glucose intolerance (35). We note that other studies have focused on the long-term effects of Fru on glucose metabolism in offspring. In our study, we found that high Fru intake also had short-term effects on the insulin concentrations and IR in offspring at 4 weeks of age.

Animal studies have shown that high Fru intake promotes GDM development and alters glucose metabolism in maternal females and offspring, although the mechanisms underlying these effects have been poorly defined. Increasing evidence indicates that IR is a chronic inflammatory process (6, 7). Inflammatory factors such as TNF-α, IL-6, and CRP have been proven to be independent risk factors for IR. Therefore, blockers or antagonists of these inflammatory factors potentially can be used to treat T2DM (36). In a human population study, the concentrations of IL-6 and IL-1β were significantly higher in patients with GDM than in normal subjects (37). Numerous studies have focused on the effect of high Fru intake on inflammation, demonstrating that consumption of a high-Fru diet induces inflammation in the heart, liver, kidney, and central nervous system (38, 39). In a study of male rats, an 8-week exposure to 20% Fru (w/v) in drinking water led to significant increases in the expression of IL-6 and TNF-α in the liver, which was accompanied by obvious steatosis and pathological features of inflammation (40). In mice with Fru-induced uric acid nephropathy, activation of the NF-κB signaling pathway led to the increased expression of TNF-α and IL-1β (41). Similarly, an *in vivo* study showed that consumption of a high-Fru diet (60%) led to a significant increase in plasma IL-1β concentrations in male offspring (42). Consistent with those findings, we showed that high Fru intake promoted inflammation in maternal mice during pregnancy and at 4 weeks postpartum, as well as in their offspring.

Nuclear factor-κB, an essential transcription factor, regulates the secretion of inflammatory factors and initiates or amplifies inflammatory responses. The activation of NF-κB is the central link in the inflammatory response chain, and this link can induce the synthesis of NLRP3 and pro-IL-1β, the first step toward activation of the NLRP3 inflammasome (43). The NLRP3 inflammasome is the most well studied inflammasome and has been shown to be linked closely to various inflammatory diseases. For example, T2DM is an energy metabolism disorder associated with chronic inflammation, which in turn is a manifestation of IR. Zhou et al. showed for the first time that *Nlrp3* knockout mice fed a high-fat diet exhibited significantly improved IR compared with control mice (12). Yun hee youm et al. also showed higher serum insulin concentrations in obese *Nlrp3* and *Pycard* (encodes ASC) knockout mice than in wild-type mice (control group) (44). In an *in vitro* study, a Fru-induced decrease in micro-RNA 200a expression in BRL-3A cells was associated with activation of the NLRP3 inflammasome and dysregulation of lipid metabolism-related proteins, leading to inflammation, and intracellular lipid deposition (45). In a rat study, treatment with 10% Fru for 6 weeks induced hyperuricemia, IR, and renal inflammation via activation of the NLRP3 inflammasome and the TLR4–MyD88 signaling pathway (46). In our study, we found that treatment with 20% Fru in drinking water before and during gestation-induced activation of NF-κB and the NLRP3 inflammasome, resulting in IR in maternal and offspring mice.

In conclusion, we have shown that high Fru intake before and during gestation can induce inflammation and IR in maternal mice during and up to 4 weeks postpartum and also can induce these effects in the offspring. Furthermore, Fru intake may promote IR via activation of the NF-κB–NLRP3 signaling pathway. Our experiments have proven the adverse effects of a Fru-rich regular diet on glucose metabolism in maternal animals during and after pregnancy and in their offspring.

## Data Availability Statement

The raw data supporting the conclusions of this article will be made available by the authors, without undue reservation.

## Ethics Statement

The animal study was reviewed and approved by Medical Ethics Committee of Shenzhen Nanshan District People's Hospital.

## Author Contributions

GD developed the overall research plan and oversaw the study. YL and YW designed and conducted the study, and completed the writing of the manuscript. XL and LW helped the animal experiments. HC and RS helped the data processing. SS helped the manuscript revision. The authors accept full responsibility for the design and conduct of the study, have access to the data and controlled the decision to publish. All authors contributed to the article and approved the submitted version.

## Funding

This study was supported by the National Natural Science Foundation of China (grant number 82103821), the Shenzhen Science and Technology Innovation Committee (grant numbers JCYJ20190809102203602 and JCYJ20210324112400002), and the Guangdong Basic and Applied Basic Project (grant number 2019A1515110456).

## Conflict of Interest

The authors declare that the research was conducted in the absence of any commercial or financial relationships that could be construed as a potential conflict of interest.

## Publisher's Note

All claims expressed in this article are solely those of the authors and do not necessarily represent those of their affiliated organizations, or those of the publisher, the editors and the reviewers. Any product that may be evaluated in this article, or claim that may be made by its manufacturer, is not guaranteed or endorsed by the publisher.

## Abbreviations

ASC: Apoptosis-associated speck-like protein containing caspase recruitment domain
Caspase-1: Cysteinyl aspartate specific proteinase-1
CRP: C-reactive protein
FBG: Fasting blood glucose
FINS: Fasting serum insulin
Fru: Fructose
HFCS: High-fructose corn syrup
HOMA-IR: Homeostasis model of assessment for IR index
HOMA-ISI: Homeostasis model of assessment for insulin sensitivity index
HOMA-β%: Homeostasis model of assessment for islet β-cell function
GDM: Gestational Diabetes Mellitus
IL-1β: Interleukin-1β
IL-6: Interleukin-6
IL-17: Interleukin-17
IR: Insulin resistance
ITT: Insulin tolerance tests
LPS: Lipopolysaccharide
NF-κB p65: Nuclear factor-κB p65
NLRP3: Nucleotide binding oligomerization domain-like receptors
OGTT: Oral glucose tolerance tests
T2DM: Type 2 diabetes
TNF-α: Tumor necrosis factor α.
