# Supplementary material for: Fructose Induces Insulin Resistance of Gestational Diabetes Mellitus in Mice via the NLRP3 Inflammasome Pathway
Source: Front Nutr. 2022 Apr 12;9:839174. doi: 10.3389/fnut.2022.839174 (PMC9040551; doi:10.3389/fnut.2022.839174)
Supplement: Supplementary file 1 [file Table_1.docx]

**Supplemental Table.1 Primer Sequences used for qRT-PCR**

| Genes | Primer Sequences (5’→3’) |
| --- | --- |
| NLRP3 | F: ATGCCAGGAAGACAGCATTG |
|  | R: TCATCGAAGCCGTCCATGAG |
| IL-6  IL-17  TNF-α  CRP  IL-1β | F: GTCCTTCAGAGAGATACAGAAACT  R: AGCTTATCTGTTAGGAGACCATTG  F: TCAGCGTGTCCAAACACTGAG  R: CGCCAAGGGAGTTAAAGACTT  F: GCTCTTCTGTCTACTGAACTTCGG  R: ATGATCTGAGTGTGAGGGTCTGG  F: TTCCCAAGGAGTCAGATACTTCC  R: TCAGAGCAGTGTAGAAATGGAGA  F: TTCAGGCAGGCAGTATCACTC  R: GAAGGTCCACGGGAAAGACAC |
