# Supplementary figures and images for: Fructose Induces Insulin Resistance of Gestational Diabetes Mellitus in Mice via the NLRP3 Inflammasome Pathway
Source: Front Nutr. 2022 Apr 12;9:839174. doi: 10.3389/fnut.2022.839174 (PMC9040551; doi:10.3389/fnut.2022.839174)

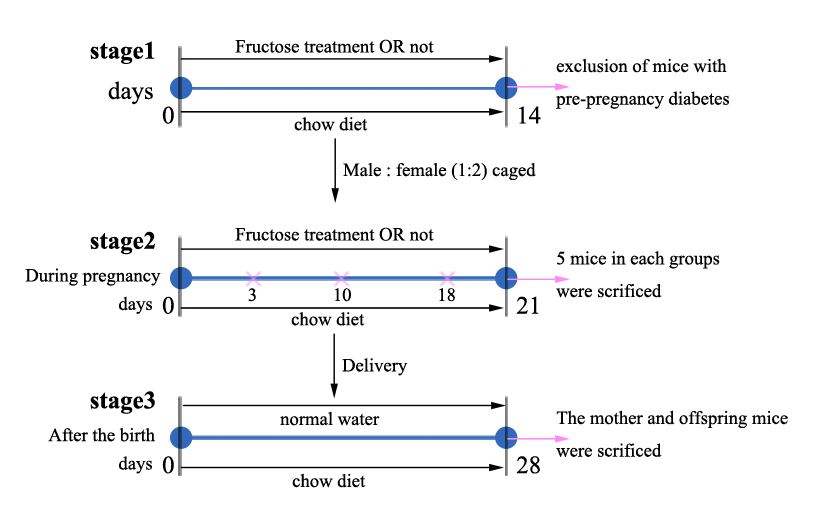

Supplement: Supplementary file 2 [file Image_1.JPEG]

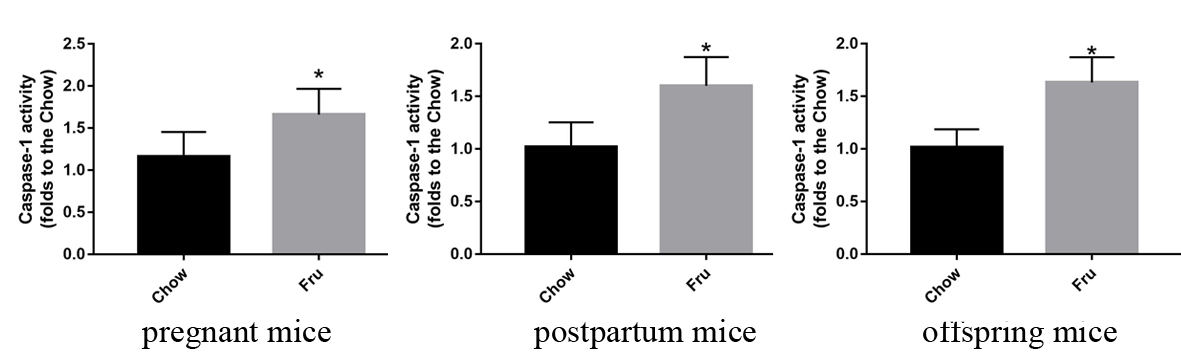

Supplement: Supplementary file 3 [file Image_2.JPEG]
